# Left ventricular unloading via percutaneous assist device during extracorporeal membrane oxygenation in acute myocardial infarction and cardiac arrest

**DOI:** 10.1177/03913988241254978

**Published:** 2024-06-10

**Authors:** Jake M Kieserman, Ivan A Kuznetsov, Joseph Park, James W Schurr, Omar Toubat, Salim Olia, Christian Bermudez, Marisa Cevasco, Joyce Wald

**Affiliations:** 1 Department of Medicine, Hospital of the University of Pennsylvania, Philadelphia, PA, USA; 2 Division of Cardiovascular Surgery, Perelman School of Medicine, University of Pennsylvania, Philadelphia, PA, USA; 3 Division of Cardiovascular Medicine, Perelman School of Medicine, University of Pennsylvania, Philadelphia, PA, USA

**Keywords:** Cardiac assist & artificial heart, cardiogenic shock, mechanical circulatory support, VA-ECMO, Impella, myocardial infarction

## Abstract

**Introduction:**

A feared complication of an acute myocardial infarction (AMI) is cardiac arrest (CA). Even if return of spontaneous circulation is achieved, cardiogenic shock (CS) is common. Venoarterial extracorporeal membrane oxygenation (VA-ECMO) supports patients with CS and is often used in conjunction with an Impella device (2.5 and CP) to off-load the left ventricle, although limited evidence supports this approach.

**Methods:**

The goal of this study was to determine whether a mortality difference was observed in VA-ECMO alone versus VA-ECMO with Impella (ECPELLA) in patients with CS from AMI and CA. A retrospective chart review of 50 patients with AMI-CS and CA and were supported with VA-ECMO (*n* = 34) or ECPELLA (*n* = 16) was performed. The primary outcome was all-cause mortality at 6-months from VA-ECMO or Impella implantation. Secondary outcomes included in-hospital mortality and complication rates between both cohorts and intensive care unit data.

**Results:**

Baseline characteristics were similar, except patients with ST-elevation myocardial infarction were more likely to be in the VA-ECMO group (*p* = 0.044). The ECPELLA cohort had significantly worse survival after VA-ECMO (SAVE) score (*p* = 0.032). Six-month all-cause mortality was not significantly different between the cohorts, even when adjusting for SAVE score. Secondary outcomes were notable for an increased rate of minor complications without an increased rate of major complications in the ECPELLA group.

**Conclusions:**

Randomized trials are needed to determine if a mortality difference exists between VA-ECMO and ECPELLA platforms in patients with AMI complicated by CA and CS.

## Introduction

Over 800,000 people suffer an acute myocardial infarction (AMI) per year in the United States.^1^ Despite modern revascularization strategies, 10% of patients develop cardiogenic shock (CS), resulting in systemic hypoperfusion and end organ dysfunction.^2,3^ Cardiac dysfunction post-AMI may result in impaired ventricular contraction, leading to decreased cardiac output, decreased coronary perfusion, and hemodynamic instability.^2,4,5^ Furthermore, myocardial injury may continue following the initial insult as the infarct extends circumferentially toward the subepicardial region causing greater decline in myocardial function.^6^ Over 50% of AMI-CS patients may experience a cardiac arrest (CA), either as a preceding event or as sequalae of CS itself, with in-hospital mortality rates as high as 60%.^4,7,8^

Patients with AMI-CS often require additional hemodynamic support with pharmacotherapy or temporary mechanical circulatory support (tMCS) both prior to and during revascularization.^9,10^ Patients may also remain in CS despite successful revascularization.^9^ Thus, the management of these patients poses a formidable challenge, demanding innovative strategies to improve survival.

While inotropes and vasopressors remain a staple of therapy in CS, the past two decades have seen increases in the use of tMCS including venoarterial extracorporeal membrane oxygenation (VA-ECMO).^11^ By providing systemic oxygenated blood flow, organ perfusion is supported irrespective of intrinsic cardiac function.^12^ While VA-ECMO decreases myocardial work in CS patients, retrograde arterial flow increases left ventricular (LV) afterload with deleterious effects on LV recovery.^12–15^ Numerous unloading strategies have been deployed to attenuate the effects of VA-ECMO on LV afterload, with the goal of improving myocardial recovery and survival.^16^

One common unloading strategy in patients on VA-ECMO is concomitant use of the Impella device.^16^ The Impella catheter (Abiomed) is an microaxial pump that traverses the aortic valve and provides continuous blood flow from the left ventricle into the aorta, thus decreasing LV afterload and myocardial work.^11,17^ Prior studies demonstrated a potential mortality benefit with VA-ECMO with simultaneous Impella (ECPELLA) compared to VA-ECMO alone.^13,18,19^ However, these studies focused on all causes of CS, not AMI-CS with concomitant CA. Therefore, the aims of this study were to determine whether a mortality difference was observed in VA-ECMO alone versus ECPELLA in patients with AMI-CS and CA and to determine the frequency of complication rates between cohorts.

## Methods

This single-center retrospective cohort study was approved by the local institutional review board. The need for informed consent was waived given the retrospective nature of the study.

### Study design and participants

A retrospective review of all patients placed on VA-ECMO or ECPELLA between 2017 and 2022 at two tertiary care centers within the same health-system was performed. Inclusion criteria consisted of patients greater than 18-years-old with AMI-CA and CA treated with VA-ECMO or ECPELLA. AMI was diagnosed according to the fourth universal definition.^20^ CA was defined as cessation of cardiac mechanical activity evidenced by an absence of signs of circulation.^21,22^ Patients who were cannulated for extracorporeal cardiopulmonary resuscitation (eCPR) were also included. Determination of tMCS strategy was made via multidisciplinary Shock Team consisting of interventional cardiologists, advanced heart failure specialists, intensivists, and cardiothoracic surgeons. The definition of shock included: (1) systolic blood pressure <90 mmHg without inotropes or need of inotropes/vasopressors to maintain systolic blood pressure >90 mmHg; (2) pulmonary capillary wedge pressure >18 mmHg; (3) central venous pressure >15 mmHg; (4) cardiac index <2.2 L/min/m^2^. If no invasive hemodynamic monitoring was available, then clinical parameters of shock included: (1) persistently elevated lactate (>2 mmol/L over 2 h) despite need for inotrope/vasopressor support; (2) signs of systemic/pulmonary overload; (3) poor end-organ perfusion (e.g. cool/mottled extremities, oliguria/acute kidney injury, and liver function test abnormalities). The decision to wean a patient from VA-ECMO or ECPELLA was made by the Shock Team.

### Study variables

Baseline demographic characteristics and clinical data were collected via the electronic health record. To characterize the varying levels of acuity between cohorts, both the *survival after veno-arterial ECMO* (SAVE) and *vasoactive ionotropic score* (VIS) was calculated for each patient.^23,24^

### Clinical endpoints

The primary outcome was 6-month mortality from initial cannulation of VA-ECMO cannulation or Impella implantation. Secondary outcomes were in-hospital mortality, complication rates, and intensive care unit data. Complication rates were calculated as both binary outcomes and per patient week (censored at time of device removal). Complications included intracranial bleeding, ischemic stroke, hypoxic brain injury, pericardial tamponade/effusion, access site ischemic event, cannula site infection, sepsis, bowel ischemia/compartment syndrome, hemolysis (lactate dehydrogenase above 1,000 U/l), arrhythmias, acute kidney injury (baseline creatine increase of 0.3 mg/dL), new renal replacement therapy, and mechanical ventilation duration. The bleeding academic research consortium (BARC) definition was used to define bleeding severity.^25^

### Management of mechanical circulatory support

All patients underwent peripheral cannulation with a standard VA-ECMO circuit consisting of venous inflow cannula, centrifugal pump and oxygenator, and arterial outflow cannula. Patients were intubated and sedated prior to cannulation. To prevent limb ischemia, a distal perfusion cannula was often placed at time of VA-ECMO cannulation. At our institution, distal perfusion cannulas are placed in the superficial femoral artery percutaneously, and the decision to place a distal perfusion cannula at the time of cannulation is under discretion of the attending physician. However, all VA-ECMO patients were monitored with continual monitoring of near-infrared spectroscopy and regular doppler pulses. A distal perfusion cannula was subsequently placed for any evidence of lower extremity perfusion loss if not placed during time of cannulation.

For the ECPELLA group, either the Impella 2.5^®^ (Abiomed) or Impella CP^®^ (Abiomed) was utilized. In a few cases, the Impella devices were placed at outside institutions. Devices implanted at our centers were placed via the femoral artery and then guided into the LV across the aortic valve. Impella device speeds were optimized under discretion of the Shock Team with the goal of minimizing hemolysis, suction events, catheter thrombosis, and malpositioning. Pump position was checked with daily chest X-rays and device repositioning was performed under echocardiographic guidance.

Both VA-ECMO and Impella 2.5^®^ (Abiomed) or Impella CP^®^ (Abiomed) support were weaned as signs of shock diminished and myocardial function improved. Typically, VA-ECMO was removed before the Impella device. The decision to explant devices was made at the discretion of the Shock team.

### Statistical analysis

Single-variable comparisons for continuous variables were made via *T*-test when normally distributed and via Wilcoxon signed-rank test when non-normally distributed. Normality was validated via the Kolmogorov-Smirnov test. Categorical variables were expressed as frequencies and comparison testing was done by Chi-squared test. When applicable, the Benjamini-Hochberg procedure was used to adjust for multiple comparisons.

In the survival analyses, univariable and multivariable Cox proportional hazards models were utilized. Visualization was done using Kaplan-Meier curves. For analysis of 6-month mortality, patients were censored at 6-months. For analysis of in-hospital mortality, patients were censored at time of discharge. Hazard ratios were extracted from corresponding models. For analysis of complication rates, odds ratios derived from logistic regression modeling were utilized to compare the relative binary occurrence of complications in each group. Incidence rate ratios were used to compare the relative difference in incidence over fixed unit time between cohorts. Analyses were performed in R.

## Results

### Participants

A total of 50 of the initial 271 reviewed patients met criteria and were included in the study. All patients had AMI-CS complicated by CA. There were 34 patients supported via VA-ECMO and 16 patients by ECPELLA. Baseline demographics were similar between groups (Table 1).

**Table 1.** Baseline demographics.

	VA-ECMO (*n* = 34)	ECPELLA (*n* = 16)	*p* Value
Age (years), median-range	55.5 (34–75)	59.0 (42–73)	0.365
Height (m), median-range	1.70 ± 0.11	1.71 ± 0.10	0.764
Weight (kg), median-range	90.40 ± 24.09	100.49 ± 22.98	0.164
Female	7 (20.6%)	3 (18.8%)	0.880
Prior CAD	8 (23.5%)	5 (31.3%)	0.562
Prior PCI	3 (8.8%)	3 (18.8%)	0.333
Prior CABG	3 (8.8%)	0 (0.0%)	0.220
Prior AMI	2 (5.9%)	3 (18.8%)	0.157
Prior HF	5 (14.7%)	6 (37.5%)	0.070
Prior PAD	2 (5.9%)	0 (0.0%)	0.322
Hyperlipidemia	14 (41.2%)	7 (43.8%)	0.863
Diabetes	14 (41.2%)	5 (31.3%)	0.500
Hypertension	19 (55.9%)	9 (56.3%)	0.981
Obesity	15 (44.1%)	8 (50.0%)	0.765
Smoking history	14 (41.2%)	11 (68.8%)	0.186
ACE-I/ARB/ARNI	8 (23.5%)	7 (43.8%)	0.165
Aspirin	7 (20.6%)	7 (43.8%)	0.102
Beta blocker	8 (23.5%)	7 (43.8%)	0.165
P2Y12 antagonist	2 (5.9%)	2 (12.5%)	0.440
Statin	9 (26.5%)	7 (43.8%)	0.249

Categorical variables are shown as counts (frequencies) and compared using a chi squared test. Continuous variables are shown as mean ± standard deviation and compared using a T-test when normally distributed and shown as median (range) and compared using a Wilcoxon Rank Sum test when nonnormally distributed.

CAD: coronary artery disease; PCI: percutaneous coronary intervention; CABG: coronary artery bypass graft; AMI: acute myocardial infarction; HF: heart failure; PAD: peripheral artery disease; ACE-I/ARB/ARNI: angiotensin converting enzyme inhibitor/angiotensin receptor blocker/angiotensin receptor/neprilysin inhibitor.

### Clinical features

**Figure 1.**
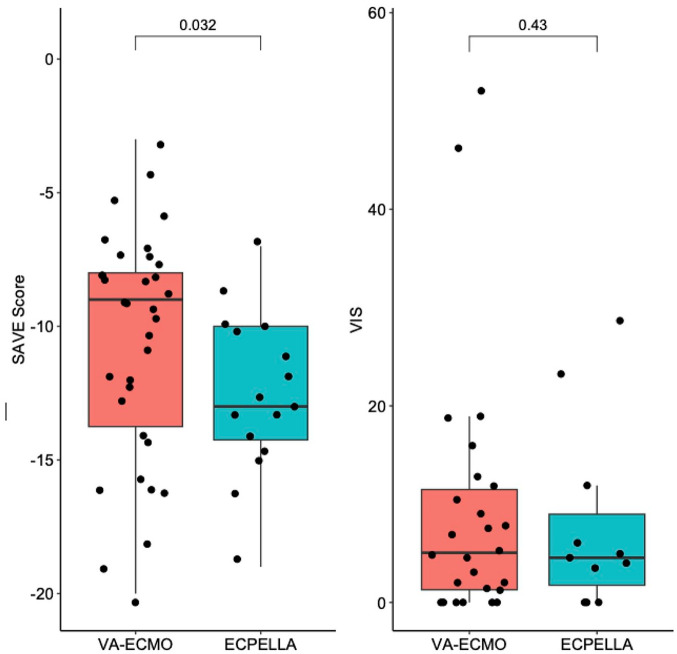
Survival After VA-ECMO (SAVE) Score and Vasoactive Inotropic Score (VIS). The SAVE score was different between groups while VIS was similar between groups. *T*-tests were used to determine statistical significance.

Table 2 summarizes CA profiles, hemodynamic/biochemical parameters, and catheterization data. Eighty-two percent of patients experienced out-of-hospital CA (79.4% VA-ECMO; 87.5% ECPELLA; *p* = 0.487). ECG findings at time of CA were variable (24% pulseless ventricular tachycardia, 24% ventricular fibrillation, 26% pulseless electric activity, and 6% asystole), but similar between groups (*p* = 0.668). Fifty-four percent of patients underwent eCPR (58.8% VA-ECMO; 43.8% ECPELLA; *p* = 0.133). Pre-ECMO CPR time was similar between groups (30 min VA-ECMO; 35 min ECPELLA; *p* = 0.983). Although 84% of patients had a STEMI prior to arrest, this differed by group (91.2% VA-ECMO; 68.8% ECPELLA; *p* = 0.044). Forty-six percent of patients had a left anterior descending culprit lesion (41.2% in VA-ECMO; 56.3% in ECPELLA; *p* = 0.414), with another 36% having a left main culprit (35.3% VA-ECMO; 37.5% ECPELLA; *p* = 1.000). Regarding biochemical data, no single parameter was statistically different between groups. However, the SAVE score (Figure 1) was significantly worse in the ECPELLA group (VA-ECMO median -9, 95% CI: -13.75 – -8; ECPELLA median -13, 95% CI: -14 – -10; *p* = 0.032). Despite this, the vasoactive-inotropic score (VIS; Figure 1) at 24 h was not significantly lower in the ECPELLA group (5.05 VA-ECMO; 4.55 ECPELLA; *p* = 0.433).

**Table 2.** Cardiac arrest, catheterization lab, and biochemical data.

	VA-ECMO (*n* = 34)	ECPELLA (*n* = 16)	*p* value
Cardiac arrest location			0.487
Out of hospital	27 (79.4%)	14 (87.5%)	
In hospital	7 (20.6%)	2 (12.5%)	
Cardiac arrest ECG			0.668
Pulseless VT	9 (26.5%)	3 (18.8%)	
Ventricular fibrillation	6 (17.6%)	6 (37.5%)	
PEA	9 (26.5%)	4 (25%)	
Asystole	2 (5.9%)	1 (6.3%)	
Other	1 (2.9%)	0 (0.0%)	
eCPR	20 (58.8%)	7 (43.8%)	0.133
Pre-ECMO CPR time (min)	30 (2–150)	35 (10–60)	0.983
ROSC prior to cannulation	11 (32.4%)	9 (56.3%)	0.147
STEMI prior to arrest	31 (91.2%)	11 (68.8%)	0.044
CAD on LHC			0.449
One vessel	10 (29.4%)	8 (50.0%)	
Two vessels	11 (32.4%)	4 (25.0%)	
Three vessels	11 (32.4%)	4 (25.0%)	
LAD culprit lesion	14 (41.2%)	9 (56.3%)	0.414
LM culprit lesion	12 (35.3%)	6 (37.5%)	1.000
Post-ROSC EF%	13.5 ± 10.3	11.6 ± 6.7	0.436
EF at time of ICU discharge	23.7 ± 18.9	15.4 ± 12.5	0.074
Early unloading (*n* = 15)	–	10 (66.7%)	–
pH (lowest)	7.23 ± 0.12	7.22 ± 0.06	0.770
Lactate (highest, mmol/L)	8.20 ± 4.49	9.85 ± 5.66	0.316
eGFR (mil/min/1.73 m^2^)	36.57 ± 29.44	25.05 ± 15.16	0.074

Categorical variables are shown as counts (frequencies) and compared using a chi squared test. Continuous variables are shown as mean ± standard deviation and compared using a *T*-test when normally distributed and shown as median (range) and compared using a Wilcoxon Rank Sum test when nonnormally distributed. Early unloading defined as placement of Impella device up to 2 h after ECMO cannulation.

VT: ventricular tachycardia; PEA: pulseless electrical activity; eCPR: extracorporeal cardiopulmonary resuscitation; ROSC: return of spontaneous circulation; STEMI: ST elevation myocardial infarction; LHC: left heart catheterization; LAD: left anterior descending; LM: left main; EF: ejection fraction; eGFR: estimated glomerular filtration rate.

### Survival

**Figure 2.**
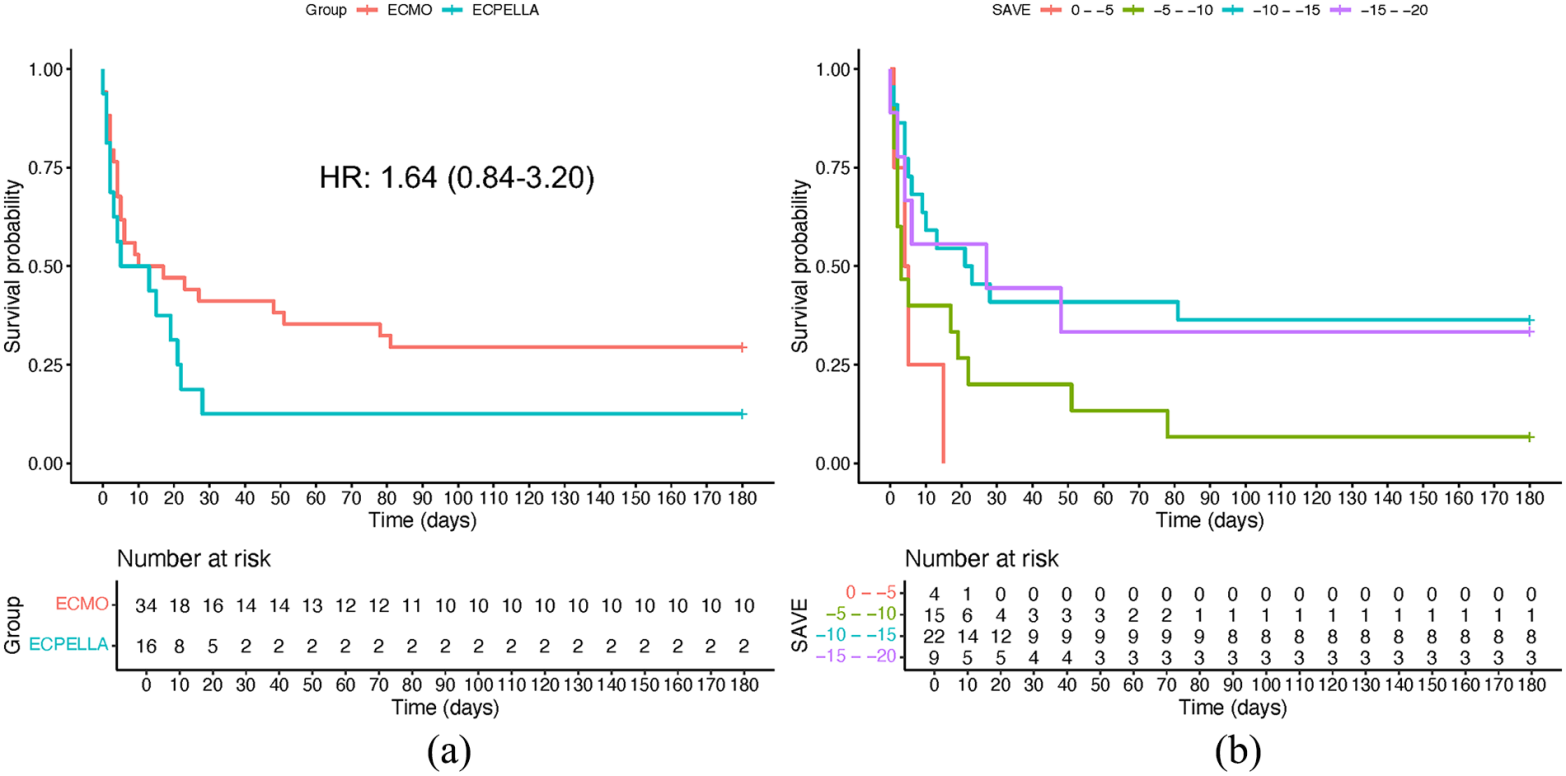
Kaplan-Meier survival curves: (a) Kaplan-Meier survival curve for ECMO and ECPELLA cohorts with associated fit hazard ratio and 95% confidence interval and (b) Kaplan-Meier survival curves of pooled cohort when stratified by presenting SAVE score demonstrate increasing mortality with decreasing SAVE scores. HR = hazard ratio.

A total of 10 patients (29.4%) in VA-ECMO and 2 patients (12.5%) in the ECPELLA group survived to 6-months (Figure 2(a)). Of the 15 ECPELLA patients where the Impella and VA-ECMO were not initiated simultaneously, 5 had the Impella placed after and 10 before VA-ECMO initiation. There was no difference in 6-month survival between those receiving the Impella prior to or after VA-ECMO initiation (OR = 2.25; *p* = 0.571). One patient in VA-ECMO cohort received a durable left ventricular assist device; no patients received a heart transplant. Based on uni-variable Cox proportional hazard model, the calculated 6-month mortality hazard ratio for ECPELLA versus VA-ECMO was 1.64 (95% CI 0.84–3.20). For in-hospital mortality, 14 (87.5**%**) ECPELLA and 23 (67.6%) VA-ECMO patients died during the index hospitalization, yielding an in-hospital mortality hazard ratio of 1.58 (0.81–3.08) for ECPELLA versus VA-ECMO (Supplemental Figure 1). Inclusion of the SAVE score into a bi-variable hazard model yielded an adjusted hazard ratio of 1.17 (95% CI: 0.59–2.35; Table 3). A multivariable-Cox proportional hazard’s model which accounted for all analyzed variables yielded an adjusted hazard ratio of 1.02 (95% CI: 0.16–6.64; Table 3). Factors significantly associated with mortality included SAVE score, presence of previous AMI, ejection fraction after recovery of spontaneous circulation, concomitant presence of systemic inflammatory response syndrome, obesity, smoking history, initial/peak lactate, presenting creatinine, and lowest pH during intervention. Of these, SAVE score demonstrated the greatest effect modification. Neither the uni-variable or adjusted multiple-variable hazard ratios demonstrated a significant difference between VA-ECMO and ECPELLA.

**Table 3.** Adjusted HR for significant factors.

	HR	95% CI
Unadjusted	1.64	0.84–3.20
SAVE score	1.17	0.59–2.35
Acute myocardial infarction	1.34	0.64–2.78
EF after ROSC	1.67	0.85–3.26
SIRS	2.33	1.15–4.74
Initial lactate	1.49	0.74–2.99
Highest lactate	1.31	0.63–2.71
Initial creatinine	1.71	0.87–3.34
Lowest pH	1.60	0.82–3.12
Obesity	1.67	0.85–3.25
Smoking history	1.66	0.85–3.24
Composite	1.02	0.16–6.64

HR: hazard ratio; CI: confidence interval; EF: ejection fraction; ROSC: return of spontaneous circulation; SIRS: systemic inflammatory response syndrome.

As noted previously, the ECPELLA group had worse SAVE scores (but not VIS) than the VA-ECMO groups, with ECPELLA patients tending to have SAVE scores ranging between −10 and −15, as compared to the VA-ECMO groups, which clustered between −5 and −10 (Figure 1). As expected, lower SAVE scores corresponded to increased mortality (Figure 2(b)).

### Complication rates

**Figure 3.**
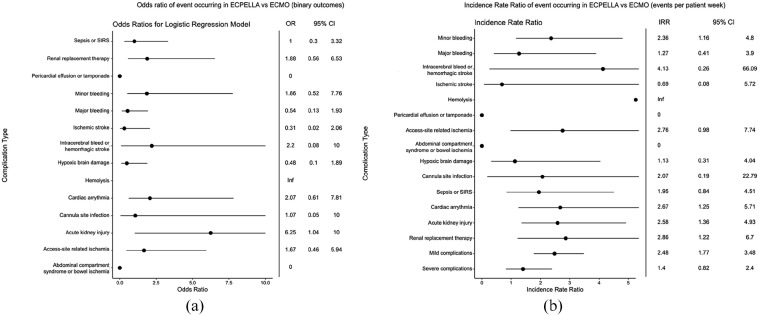
Complication rates in ECPELLA versus ECMO: (a) Logistic-regression model fit odds ratios (OR) for studied complications for ECPELLA versus ECMO. Outcomes were considered as binary, that is if at least one of the specified complications occurred during a patient’s hospitalization, (b) Incidence rate ratio (IRR) of complication occurrence for ECPELLA versus ECMO, as measured by the ratio of events per patient week. Note that an IRR/OR of 0 indicates that none of the given complication occurred in the ECPELLA group while an IRR/OR of infinity indicates that the complication only occurred in the ECPELLA cohort. OR = odds ratio; CI = confidence interval; IRR = incidence rate ratio.

A list of evaluated complications is shown in Supplemental Table 1. Complications were further divided for purposes of pooled analysis into minor (minor bleeding, hemolysis, access site-related ischemia, cannula site infection, sepsis/SIRS, cardiac arrhythmias, and acute kidney injury) and severe (significant bleeding, strokes, abdominal compartment syndrome or bowel ischemia, hypoxic brain damage, and renal replacement therapy need). With regard to neurologic outcomes, overall, 18 (53%) of VA-ECMO patients and 5 (31%) of ECPELLA patients had a neurologic event, with no statistically significant difference in the frequency of each of the following types of neurologic events between both cohorts: intracranial bleeds (3% VA-ECMO vs 6% ECPELLA, *p* = 0.542), ischemic stroke (18% VA-ECMO vs 6% ECPELLA, *p* = 0.406), and hypoxic brain damage (32% VA-ECMO vs 19% ECPELLA, *p* = 0.501). While there were no significant differences in any individual complications between the ECEPLLA and VA-ECMO cohorts (Figure 3(a)), differences were observed in terms of incidence rate ratio (Figure 3(b)). The ECPELLA cohort showed a higher incidence rate of minor bleeding (IRR 2.36; 95% CI 1.16–4.80), arrhythmias (IRR 2.67; 95% CI 1.25–5.71), acute kidney injury (IRR 2.58; 95% CI 1.36–4.93) and need for renal replacement therapy (IRR 2.86; 95% CI 1.22–6.7). Overall ECPELLA, relative to VA-ECMO, had an increased rate of minor complications (IRR 2.48; 95% CI 1.77–3.48) without an increased rate of severe complications (IRR 1.4; 95% CI 0.82–2.4).

## Discussion

To our knowledge, this study is the first to directly compare VA-ECMO and ECPELLA in patients with AMI-CS who concomitantly sustained a CA. This marks a pivotal point, as our population may represent some of most critically ill cohorts to be compared in the context of VA-ECMO versus ECPELLA. Schmidt et al.^23^ demonstrated that a SAVE score of −9 to −5 conferred an in hospital survival probability of 30% or less, while a score less than or equal to −10 was associated with an in-hospital survival rate of 18% or less. The SAVE score was further validated by Chen et al.^26^ in which they found the SAVE score to be an independent predictor of mortality in patients on VA-ECMO. The mortality rates in our VA-ECMO and ECPELLA cohorts were 29.4% and 12.5%, respectively, which correlate with the median SAVE scores in each cohort.

Despite advances in cardiac clinical care, CS shock still confers incredibly high in-hospital mortality rates.^4,7,8^ Other forms of tMCS including Tandem heart (Cardiac Assist, Pittsburgh PA), intra-aortic balloon pump, and Impella without VA-EMCO involvement have not demonstrated mortality benefits in refractory CS in randomized controlled trials.^27–31^ Although there are no randomized trials directly comparing VA-ECMO and ECPELLA, there have been numerous retrospective studies that have shown mortality benefits favoring ECPELLA.^12,13,18,19^ A large meta-analysis by Russo et al.^16^ demonstrated that LV unloading with an Impella device for patients on VA-ECMO decreases mortality when compared with VA-ECMO alone. Given the results of these studies, LV unloading with the ECPELLA platform seems like a plausible strategy for treating refractory CS.

Although our study did not show a significant difference in mortality between the VA-ECMO and ECPELLA cohorts, this may be explained for a few reasons. As previously mentioned, the survival rates for both groups were incredibly low, which may limit the ability to detect survival differences between VA-ECMO and ECPELLA cohorts. Second, because only 50 patients met inclusion criteria, the power of our study was limited. Third, our ECPELLA population was likely sicker than our VA-ECMO population, as identified by a statistically significantly worse median SAVE score, which may have altered seeing a potential benefit from the Impella. Lastly, it should also be noted that this study occurred prior to use of the Impella 5.5 as an unloading strategy at our institution. The Impella 5.5 can provide more unloading relative to the Impella 2.5 and CP devices, so the ECPELLA with an Impella 5.5 may be of consideration in the future.^32^

In our study, we found that there was an increase in the incidence of minor complications without an increase in major complications between ECPELLA and VA-ECMO cohorts. Previous studies have shown significantly increased rates of hemolysis, severe bleeding, renal replacement, and access site complications in ECPELLA patients compared to VA-ECMO alone patients, which generally aligns with our observations.^13,18^ The most prevalent severe complication with increased incidence in the ECPELLA group was need for renal replacement therapy, consistent with the observed higher rate of acute kidney injury with ECPELLA. Overall, this latter observation may help guide patient selection for ECPELLA and may also aid in guiding how selective clinicians are with exposing these patients to nephrotoxins. Despite the increased rate of minor complications with ECPELLA, the lack of an increased rate of major complications is a significant insight, as it suggests that ECPELLA may have an acceptable safety profile relative to VA-ECMO as long as a center can comfortably manage minor complications. We hope that future studies can further investigate ECPELLA-associated complications to explore whether the observed lack of difference in terms of major complications is secondary to lack of power given the relatively small sample size studied here.

## Limitations

Our study has several limitations. First, our patient population is small thus limiting the power of our analysis. Despite covariate adjustment, the absence of randomization limits the potential to perform a true comparison of similar cohorts. Additionally, patients with CA have a high mortality independent of intervention, decreasing the feasibility to show survival benefit. Furthermore, there may be inherent biases in which patients are started on ECPELLA due to Shock Team preference. Lastly, as a retrospective analysis we are limited in our ability to control for sources of bias.

## Conclusions

In this limited retrospective study, there does not seem to be a mortality benefit with the addition of Impella 2.5 and CP to VA-ECMO in a high-risk post AMI group who sustained a concomitant CA. The observation that the cohort receiving an adjunctive Impella was sicker may have limited the ability to show a positive result. While there was an increase in the incidence of minor complications in the ECPELLA cohort, there was no difference in major complications. Future randomized controlled trials comparing ECPELLA versus VA-ECMO are required to further elucidate if the addition of Impella, especially with the Impella 5.5, to VA-ECMO in AMI with CS with concomitant CA provides a mortality benefit.

## Supplemental Material

sj-pdf-1-jao-10.1177_03913988241254978 – Supplemental material for Left ventricular unloading via percutaneous assist device during extracorporeal membrane oxygenation in acute myocardial infarction and cardiac arrest Supplemental material, sj-pdf-1-jao-10.1177_03913988241254978 for Left ventricular unloading via percutaneous assist device during extracorporeal membrane oxygenation in acute myocardial infarction and cardiac arrest by Jake M Kieserman, Ivan A Kuznetsov, Joseph Park, James W Schurr, Omar Toubat, Salim Olia, Christian Bermudez, Marisa Cevasco and Joyce Wald in The International Journal of Artificial Organs
