# Supplementary material for: Left ventricular unloading via percutaneous assist device during extracorporeal membrane oxygenation in acute myocardial infarction and cardiac arrest
Source: Int J Artif Organs. 2024 Jun 10;47(6):401–10. doi: 10.1177/03913988241254978 (PMC11826357; doi:10.1177/03913988241254978)
Supplement: sj-pdf-1-jao-10.1177_03913988241254978 – Supplemental material for Left ventricular unloading via percutaneous assist device during extracorporeal membrane oxygenation in acute myocardial infarction and cardiac arrest [file sj-pdf-1-jao-10.1177_03913988241254978.pdf]

Supplemental Table 1. Post-Procedural Complications

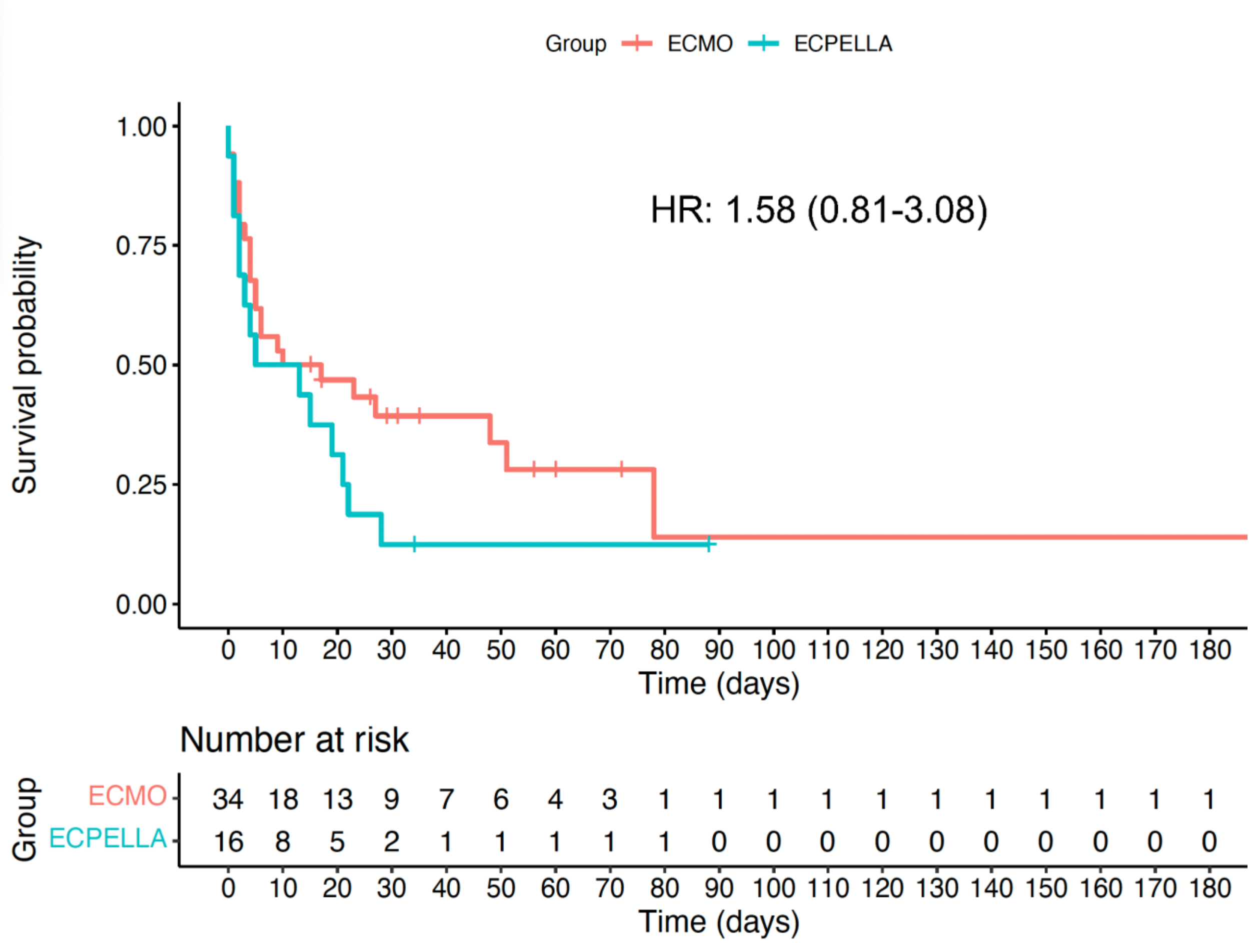

Supplementary Figure 1. Kaplan-Meier survival curve for in-hospital mortality for ECMO and ECPELLA cohorts with associated fit hazard ratio and 95% confidence interval. Patients were censored at time of hospital discharge. HR = hazard ratio.

	VA-ECMO (n=34)	ECPELLA (n=16)	P value
BARC<br>3a<br>3b<br>4<br>5a<br>5b	 20 (58.8%)<br>11 (32.4%)<br>1 (2.9%)<br>0 (0.0%)<br>1 (2.9%)	 11 (68.8%)<br>4 (25%)<br>0 (0.0%)<br>0 (0.0%)<br>0 (0.0%)	0.495
Total pRBCs	6 (4-22)	10 (3-26)	0.773
Intracranial Bleed	1 (2.9%)	1 (6.3%)	0.542
Ischemic Stroke	6 (17.6%)	1 (6.3%)	0.406
Hemolysis	0 (0.0%)	1 (6.3%)	0.320
Pericardial Effusion/Tamponade	6 (17.6%)	0 (0.0%)	0.159
Access Site Ischemic Event	9 (26.5%)	6 (37.5%)	0.427
Bowel Ischemia/Compartment syndrome	3 (8.8%)	0 (0.0%)	0.542
Hypoxic Brain Damage	11 (32.4%)	3 (18.8%)	0.501
Cannula Site Infection	2 (5.9%)	1 (6.3%)	1.00
Sepsis	17 (50.0%)	8 (50.0%)	1.00
Arrythmia	17 (50.0%)	11 (68.8%)	0.253
AKI	24 (70.6%)	15 (93.8%)	0.065
KRT	13 (38.2%)	9 (56.3%)	0.306
Mechanical Ventilation Duration (min)	12960 (7200, 23040)	21600 (7200, 30240)	0.558
CPC (1-5)	5 (3,5)	5 (5,5)	0.223

Categorical variables are shown as counts (frequencies) and compared using a chi squared test. Continuous variables are shown as a median (range) and compared using a Wilcoxon Rank Sum test when nonnormally distributed. P values adjusted by Benjamini -Hochberg method for multiple comparisons. BARC indicates bleeding academic research consortium; pRBCs, packed red blood cells; AKI, acute kidney injury; KRT, kidney replacement therapy; CPC, cerebral performance category.
